# Quantitative Proteomics Reveal Region-Specific Alterations in Neuroserpin-Deficient Mouse Brain and Retina: Insights into Serpini1 Function

**DOI:** 10.3390/proteomes12010007

**Published:** 2024-03-14

**Authors:** Shahab Mirshahvaladi, Nitin Chitranshi, Ardeshir Amirkhani, Rashi Rajput, Devaraj Basavarajappa, Roshana Vander Wall, Dana Pascovici, Angela Godinez, Giovanna Galliciotti, Joao A. Paulo, Veer Gupta, Stuart L. Graham, Vivek Gupta, Mehdi Mirzaei

**Affiliations:** 1 Macquarie Medical School, Macquarie University, Sydney, NSW 2109, Australia; seyed-shahab-oddin.mirshahv@hdr.mq.edu.au (S.M.); nitin.chitranshi@mq.edu.au (N.C.); rashi.rajput@hdr.mq.edu.au (R.R.); devaraj.basavarajappa@mq.edu.au (D.B.); roshana.vanderwall@mq.edu.au (R.V.W.); angela.godinez@hdr.mq.edu.au (A.G.); stuart.graham@mq.edu.au (S.L.G.); vivek.gupta@mq.edu.au (V.G.); 2 Australian Proteome Analysis Facility, Macquarie University, Sydney, NSW 2109, Australia; ardeshir.amirkhani@mq.edu.au (A.A.); dana.pascovici@mq.edu.au (D.P.); 3 Institute of Neuropathology, University Medical Center Hamburg-Eppendorf, 20246 Hamburg, Germany; 4 Department of Cell Biology, Blavatnik Institute, Harvard Medical School, Boston, MA 02115, USA; joao_paulo@hms.harvard.edu; 5 School of Medicine, Deakin University, Geelong, VIC 3125, Australia; veer.gupta@deakin.edu.au

**Keywords:** neuroserpin, neurodegeneration, quantitative proteomics, neuroprotection, tandem mass tags

## Abstract

Neural regeneration and neuroprotection represent strategies for future management of neurodegenerative disorders such as Alzheimer’s disease (AD) or glaucoma. However, the complex molecular mechanisms that are involved in neuroprotection are not clearly understood. A promising candidate that maintains neuroprotective signaling networks is neuroserpin (Serpini1), a serine protease inhibitor expressed in neurons which selectively inhibits extracellular tissue-type plasminogen activator (tPA)/plasmin and plays a neuroprotective role during ischemic brain injury. Abnormal function of this protein has been implicated in several conditions including stroke, glaucoma, AD, and familial encephalopathy with neuroserpin inclusion bodies (FENIB). Here, we explore the potential biochemical roles of Serpini1 by comparing proteome changes between neuroserpin-deficient (NS^−/−^) and control mice, in the retina (RE), optic nerve (ON), frontal cortex (FC), visual cortex (VC), and cerebellum (CB). To achieve this, a multiple-plex quantitative proteomics approach using isobaric tandem mass tag (TMT) technology was employed followed by functional enrichment and protein–protein interaction analysis. We detected around 5000 proteins in each tissue and a pool of 6432 quantified proteins across all regions, resulting in a pool of 1235 differentially expressed proteins (DEPs). Principal component analysis and hierarchical clustering highlighted similarities and differences in the retina compared to various brain regions, as well as differentiating NS^−/−^ proteome signatures from control samples. The visual cortex revealed the highest number of DEPs, followed by cerebellar regions. Pathway analysis unveiled region-specific changes, including visual perception, focal adhesion, apoptosis, glutamate receptor activation, and supramolecular fiber organization in RE, ON, FC, VC, and CB, respectively. These novel findings provide comprehensive insights into the region-specific networking of Serpini1 in the central nervous system, further characterizing its potential role as a neuroprotective agent. Data are available via ProteomeXchange with identifier PXD046873.

## 1. Introduction

Neurodegenerative diseases are categorized by the gradual loss of neuron numbers and function and are associated with a decline in cognitive abilities and movement [1]. The number of people suffering from neurodegenerative diseases such as dementia is growing significantly, representing a critical public health problem. For example, the number of individuals affected by dementia is projected to increase to 152 million by 2050, with a disproportionate impact on developing countries [2]. The main reason is the increase in life expectancy with a rise in the number of older adults: one-third of the population will be aged over 65 by 2030 [3,4]. Despite continuous efforts by modern medicine to provide a solution, large proportions of the aging population develop neurodegenerative disorders such as Alzheimer’s disease (AD) and glaucoma [5].

The neurodegenerative mechanisms observed in these different pathologies are the result of a complex cascade, including neuroinflammation, mitochondrial dysfunction, perturbed Ca^2+^ influx, excitotoxicity, and protein aggregate accumulation [6,7]. Eventually, these insults lead to a progressive loss of synapses/axons and ultimately neuronal cell death [8]. Hence, potential therapeutic strategies involve impeding these degenerative processes to preserve neurons and their connections, in both acute (trauma or stroke) and chronic neurodegenerative disorders (dementia, glaucoma, AD) [9].

A promising candidate for neuroprotection is neuroserpin, a serine protease inhibitor (serpin) that mainly regulates tissue-type plasminogen activator (tPA)/plasmin activity in the nervous system [10]. Neuroserpin (Serpini1) is mainly involved in axonal development, synaptic plasticity, and neurite outgrowth through inhibiting tPA [11,12,13] as well as tPA-independent mechanisms [14,15]. Alteration in the expression level or activity of this protein has been reported in brains of schizophrenic patients [16], AD [17], glaucoma [18], and Sporadic Creutzfeldt–Jakob disease (sCJD) [19]. Recently, our group has demonstrated that not only is neuroserpin ablation or neutralization detrimental to the retina in a mouse model of glaucoma, but its overexpression is protective against glaucoma injury [20]. Still, the underlying mechanisms by which Serpini1 acts as a neuroprotective agent in retina and the brain are not fully understood.

Mass spectrometry (MS)-based quantitative proteomics has proved to be a crucial high-throughput tool for unbiased, precise, and reproducible protein identification/quantitation [21]. Advances in liquid chromatography-tandem MS (LC-MS/MS) have enabled the quantification of thousands of proteins in various biological samples [22]. The LC-MS/MS technique has established itself as invaluable in exploring functional pathways and mechanisms. One of the latest advances in quantitative MS proteomics is the development of the tandem mass tag (TMT) as a powerful tool to analyze changes in protein expression levels, providing a comprehensive approach to study responses to various interventions [23]. Employing the TMT technique allows for the simultaneous identification and quantification of tagged proteins across experimental conditions for up to 18 samples [24]. Therefore, TMT proteomics enable accurate identification of proteins and signaling pathways that undergo changes in the presence of neuroprotective agents, shedding light on potential therapeutic targets [25]. The ability to assess changes in protein abundance with high precision and dynamic range shifts a new paradigm in understanding cellular responses to neuroprotective stimuli, thereby contributing to mechanisms underlying neuroprotection [26,27].

Knockout mouse models in which the open reading frame of the gene is disrupted, have been the ideal tool to explore the biological function of any protein [28]. There are numerous genetic and physiological similarities between mice and humans, particularly in the central nervous system [29,30], where the mouse serves as one of the best models to study biochemical processes in the human brain. In this work, we used multiplex TMT-based quantitative proteomics to explore the effects of neuroserpin ablation on the proteome of different regions of the mouse brain and retina. Afterwards, we validated the altered expression of key proteins by Western blotting. Our results provide region-wise evidence regarding the effects of Serpini1 ablation on the nervous system in mice and its implications to better understand its potential neuroprotective mechanisms.

## 2. Materials and Methods

### 2.1. Animals

Neuroserpin-deficient mice (NS^−/−^) employed in this work were sourced from the animal facility at the University Medical Center Hamburg-Eppendorf and subsequently bred in the Central Animal Facility of Macquarie University. Briefly, NS^−/−^ mice were generated by inserting a neomycin cassette into coding exon two of Serpini1 [31,32] and backcrossed to a C57BL/6J background for at least ten generations until establishment of animal colonies. For this study, a total of five NS^−/−^ and five wildtype littermates (WT) of either sex were used. Animals were maintained in a standard environment with a constant temperature of 21 °C ± 2 °C and a 12 h light/dark cycle, with *ad libitum* access to regular laboratory chow and water, until tissue harvest. All mice were euthanized using a cervical dislocation technique, followed by transcardial perfusion [33] using normal saline. For tissue collection, the retinas (RE) and optic nerves (ON) were extracted from the eyeballs first using the “winkling” method [34]. Then, brains were removed from the skull and snap frozen in liquid nitrogen for further processing. Brains were later dissected [35] into the frontal cortex (FC), visual cortex (VC), and cerebellum(CB) and were stored at −80 °C for downstream total protein extraction.

### 2.2. Protein Sample Preparation

Frozen tissues were washed three times in cold phosphate-buffered saline (PBS) and then lysed using a 1:5 (*vol*/*vol)* ratio RIPA lysis buffer (20 mM Tris-HCl, 0.5 mM EGTA, 1% Triton X-100, 0.1% Sodium Deoxycholate, 0.1% SDS, pH 7.4) containing PhosphoSTOP™ phosphatase and cOmplete™ protease inhibitor cocktails (Roche Applied Science, Mannheim, Germany). Samples were homogenized by the bead-beating method using a FastPrep-24^®^ Classic (MP Biomedicals, Santa Ana, CA, USA) as per the manufacturer’s instructions and then sonicated using a VEVOR 3L bath sonicator (3 incubations/10 m) in a cold room. Any remaining insoluble materials were removed by centrifugation at 10,000× *g* for 10 min at 4 °C. The supernatants were transferred to new Eppendorf tubes and their protein concentration was measured by a BCA assay kit (Pierce, Rockford, IL, USA) in the linear range of the assay. The quality of protein extraction and the validity of concentration readings were further examined using SDS-PAGE.

Total proteins were reduced using 5 mM dithiothreitol (DTT) for 15 min at room temperature, and then alkylated with 10 mM iodoacetamide for 30 min in the dark. The alkylation reaction was quenched with the addition of 5 mM DTT for 15 min in the dark. Dual digestion using Trypsin/Lys-C (Thermo Scientific, Rockford, IL, USA) was carried out on 50 μg of total protein using S-Trap columns [36] with slight modifications. Initially, additional SDS was added to the protein lysates so that the final SDS concentration reached 5%. Trypsin in 100 mM TEAB solution was used as the digestion buffer at a 1:100 enzyme-to-protein (*w*/*w*) ratio overnight at room temperature in a humidified chamber. Peptides were eluted according to S-Trap protocol and vacuum centrifuged to dryness for 3 h at 45 °C.

### 2.3. Tandem Mass Tag Labelling of Peptides

**Figure 1:**
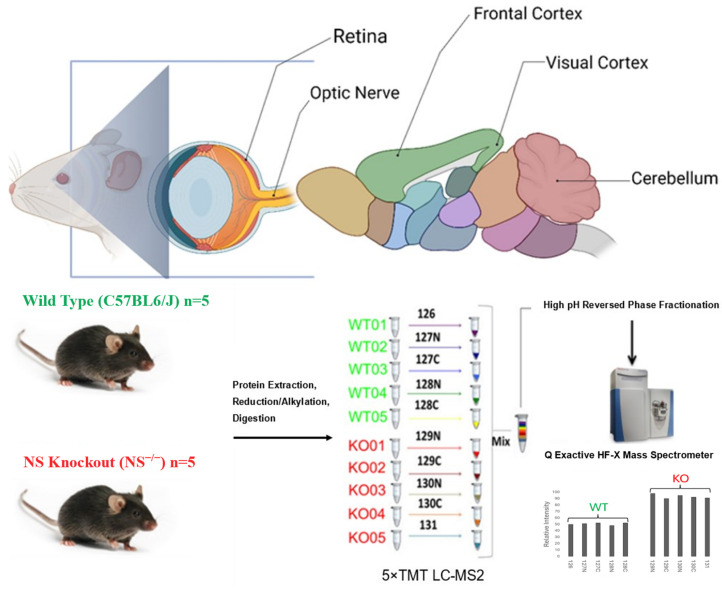
Experimental design and the TMT labeling workflow of the current study. Brain, retinas, and optic nerves from WT and NS^−/−^ mice (C57BL6/J) were harvested (n = 5). Brain tissues were further dissected into the frontal cortex, visual cortex, and cerebellum. Extracted proteins were subjected to reduction, alkylation, and subsequent digestion with Trypsin/Lys-C. Solubilized peptides were quantified and labeled using five rounds of 10-plex TMT reagent (channels 126-128: WT, channels 129-131: NS^−/−^). Labelled samples from each region were pooled together, fractionated into right fractions by high pH reversed phase chromatography, and analyzed by LC-ESI-MS/MS on a Q Exactive HFX mass spectrometer. After data normalization and filtering, functional enrichment and protein network analysis were performed using various bioinformatic tools.

Five separate 10-plex TMT experiments were designed to accommodate the retina, optic nerve, frontal cortex, visual cortex, and cerebellum of neuroserpin-deficient and wildtype animals, as outlined in Figure 1. For data acquisition, we divided these 50 samples into five sets for 10plex LC-MS2 TMT isobaric labeling. Briefly, dried peptides were resuspended in 100 mM HEPES (pH 8.2) buffer and peptide concentrations were measured using the MicroBCA protein assay kit (Thermo Scientific) and labeled with 0.8 mg of TMT per 50 μg of peptide. The labeling process was carried out at room temperature for 1 h with brief vortexing every 15 min. The reaction was quenched by the addition of 8 μL of 5% fresh hydroxylamine to each tube. For each TMT experiment, ten labeled tubes were pooled and dried for 3 h at 45 °C using a Vacufuge plus 5305 Concentrator (Eppendorf AG, Hamburg, Germany). To further increase the proteome coverage and reduce complexity, labeled peptides were fractionated into 8 fractions according to their hydrophobicity using the Pierce High pH Reversed-Phase Peptide Fractionation Kit. Next, the fractions were dried in a speed vacuum and reconstituted in 1% formic acid afterwards.

### 2.4. Liquid Chromatography-Tandem Mass Spectrometry

A Q Exactive HFX Orbitrap mass spectrometer (Thermo Scientific) coupled to an UltiMate 3000 HPLC system (Thermo Scientific) was used for proteome analysis. Solubilized peptides (1 μg) were loaded onto a peptide trap (300 μm × 5 mm C18 PepMap 100, 5 µm, 100 Å, Thermo Scientific) using 0.1% (*v*/*v*) formic acid as the loading buffer. The chromatographic separation was achieved on reverse-phase columns (75 μm × 30 cm Reprosil-Pur 120 Å C18-AQ 3µm, Dr Maisch) which were packed in-house. Separation of the labeled peptides was accomplished using a 140-min gradient of 1–30% solvent A (0.1% formic acid) and B (99.9% acetonitrile/0.1% formic acid). The mass spectrometer was set in the data-dependent acquisition (DDA) mode to automatically switch between full MS and MS/MS acquisition. Following the full MS scan within 350–1850 *m*/*z*, data-dependent MS/MS spectra were acquired at 120,000 resolution (*m*/*z* 200) with an automatic gain control (AGC) target value of 3 × 10^6^ ions. For higher-energy collisional dissociation (HCD) fragmentation, the ten most abundant precursor ions were selected with a precursor isolation window of 0.8 *m*/*z*. HCD-normalized collision energy was set to 33% and fragmentation ions were detected in the Orbitrap at a resolution of 45,000 (*m*/*z* 200). Following MS/MS selection, target ions were dynamically excluded for 30 s. The mass spectrometry proteomics data have been deposited to the ProteomeXchange Consortium (http://proteomecentral.proteomexchange.org) via the PRIDE partner repository [37] with the dataset identifier PXD046873.

### 2.5. Database Search, Peptide Quantification and Statistical Analysis

Raw data files were processed in Proteome Discoverer V2.1 (Thermo Scientific) using SEQUEST HT (Thermo Scientific) against the reviewed UniProtKB/Swiss Prot protein database of Mus musculus (release 16 February 2023). The search was performed with ±10 ppm and 0.02 Da tolerance for MS1 and a MS/MS, respectively. Carbamidomethyl (C) was set as a static modification, while TMT 6-plex (N-term, K), oxidation (M), deamidation (N, Q), Glu->pyro-Glu (N-term E), Gln->pyro-Glu (N-term Q), and acetylation (protein N terminus) were set as dynamic modifications. The percolator algorithm was used to discriminate valid peptide-spectrum matches from false positives, followed by the computation of statistics such as q-values and posterior error probabilities (PEPs). To minimize false positives and ensure accurate protein quantification, only master proteins confirmed by at least two unique peptides exhibiting a stringent False Discovery Rate (FDR) of less than 0.01 were retained for further steps.

### 2.6. Normalization of Multiplexed Quantitative Proteomics Data and Bioinformatic Analysis

Datasets were further analyzed using the TMTPrepPro analysis pipeline and combined into a single expression matrix. The TMTPrepPro scripts are implemented in the R programming language and are available as an R package (3.4.0), which was accessed through a graphical user interface provided via a local GenePattern server [38]. TMT reporter-ion intensity values were normalized by summing values across all peptides within each channel and then correcting each channel to the same totaled value. Differentially expressed proteins (DEPs) were identified using a Student’s t-test with Bonferroni multiple comparison correction (adj. *p*-value < 0.05, fold change > 1.2 or <0.83). Heatmap, volcano plot, and unsupervised principal component analysis (PCA) were generated using built-in functions in the Omics Playground pipeline [39] for assessing the clustering and batch effect in the samples. Gene Ontology (GO) enrichment analysis and pathway enrichment were performed in ShinyGO using differentially expressed proteins with an adjusted *p*-value of <0.05. Enrichment terms were computed using hypergeometric distribution followed by false discovery rate (FDR) correction [40]. Further, the protein–protein interaction network was analyzed using the stringApp [41] plugin in the Cytoscape platform. Network nodes consisting of pooled differentially expressed proteins (confidence score > 0.7) were labeled using their gene symbols, and fold changes quantified from TMT analysis were color-coded as donut bars using the Omics Visualizer app [42]. The stringApp plugin was used to assign enriched (FDR < 0.05) biological process (BP), molecular function (MF), and pathway to protein-protein clusters.

### 2.7. Protein Validation by Western Blotting

Western blotting was performed to validate the relative abundance of selected DEPs resulting from TMT proteomics analysis as described previously [43]. In brief, 20 µg of total protein was run on 10% Novex NuPage (Invitrogen, Carlsbad, CA, USA) mini gels using MOPS buffer at 150 V for 90 min and then transferred to PVDF membranes using iBlot 2 (Invitrogen) equipment and pre-assembled stacks. Membranes were blocked in Tris-buffered saline (TBS-T) (20 mM Tris-HCl, pH 7.4, 100 mM NaCl, and 0.1% Tween 20) containing 5% skimmed milk. Blots were probed with the following primary antibodies: anti-neuroserpin (ab33077, Abcam (Cambridge, MA, USA), 1:2000), anti-Vdac2 (ab15895, Abcam, 1:1000), anti-Mapt (#4019, Cell Signaling (Beverly, MA, USA), 1:2000), anti-synaptophysin (ab32127, Abcam, 1:8000), and anti-Actin (ab6276, Abcam, 1:20,000) as the loading control. Next, blots were incubated with relevant HRP-labeled secondary antibodies and the signals were detected using the Clarity™ chemiluminescence substrate (Bio-Rad, Hercules, CA, USA) on an ImageQuant LAS 4000 chemiluminescent image analyzer (GE Healthcare, Piscataway, NJ, USA) using the built-in autoexposure function and in the linear range. Band intensity quantification and background removal was carried out using the ImageJ software package (version 1.53n) [44] and in accordance with the guidelines for quantitative Western blotting [45].

### 2.8. Statistical Analysis

Quantified Western blot bands were analyzed using GraphPad Prism (v.9.1). All values here were represented as the mean ± standard error of the mean (SEM) error bars for the provided N size. Statistical analysis was performed using a one-way ANOVA followed by Bonferroni’s post hoc multiple comparisons adjustment. The statistical significance threshold value was set at *p* < 0.05.

## 3. Results

### 3.1. Differential Regulation of the Proteome in the Different Brain Regions of Neuroserpin-Deficient Mice

**Figure 2:**
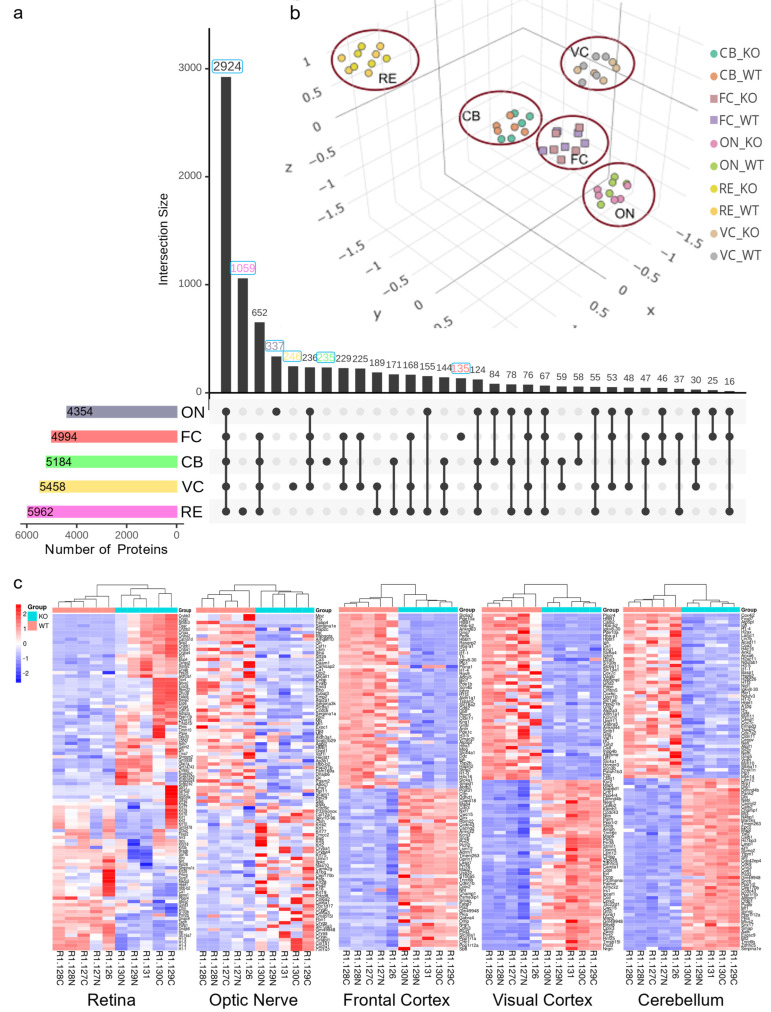
TMT proteomic analysis results and quality control steps. (**a**) Upset plot of unique and overlapping identified proteins between different regions of NS^−/−^ vs. WT mice. (**b**) Unsupervised principal components analysis (PCA), visualizing the similarity of regions for NS^−/−^ vs. WT samples. Each region formed a distinct cluster in 3D space, with the retina and visual cortex showing the most variance from others. (**c**) Heatmaps showing gene expression sorted by 2-way hierarchical clustering in NS^−/−^/WT for all the regions. Red and blue indicate relative increase or decrease in protein abundance (fold change), respectively.

The frontal cortex, visual cortex, and cerebellum dissected from 12-month-old mice brain (Figure 1) were analyzed along with the retina and optic nerves using five separate sets of 10plex LC-MS2 TMT runs. The analyses of the spectra allowed for a total quantitation of 6432 non-redundant proteins across all these regions. A total of 5962, 4354, 4994, 5458, and 5184 non-redundant proteins were identified from 12-month murine retina (RE), optic nerve (ON), frontal cortex (FC), visual cortex (VC), and cerebellum (CB), respectively (Figure 2a). The highest number of unique proteins was observed in the retina, where 1059 proteins were exclusively detected. In total, 2012 proteins were exclusively present in either region as shown in the Upset plot, while 2924 were shared between all analyzed tissues.

Using principal component analysis (PCA) across all regions, a clear separation of the retina from other brain regions and even the optic nerve (Figure 2b) was observed. Further PCA for each region suggested a distinct pattern of proteome signature between neuroserpin-deficient samples and controls, except in the optic nerve for which there were overlaps in PC1 and PC2 (Supplementary Figure S1). The quality of TMT datasets was evaluated using a series of built-in features in the TMTPrepPro pipeline and Omics Playground, such as box plots and clustering analysis, which showed no noticeable batch effect and remarkable outliers. As illustrated by the heatmaps, hierarchical clustering separated NS^−/−^ and WT samples in all the regions (Figure 2c).

**Figure 3:**
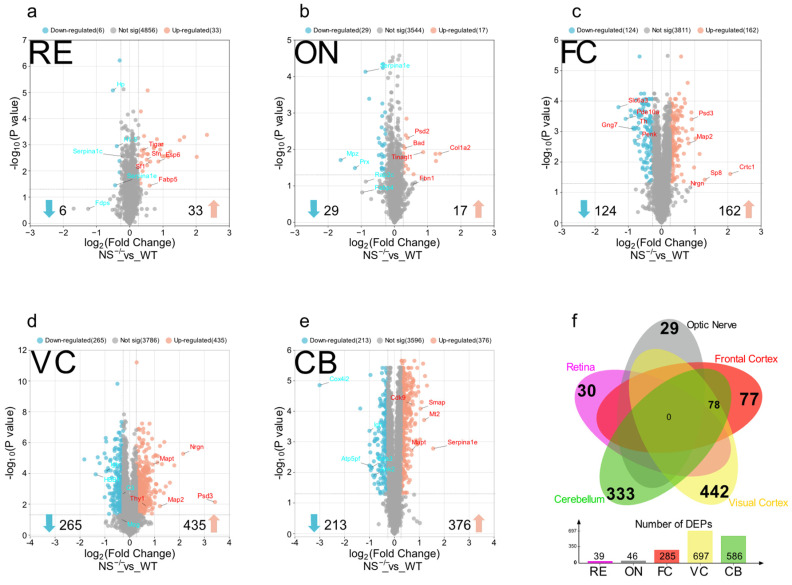
Volcano plots showing the criteria for differentially regulated proteins (DEPs) in the (**a**) retina, (**b**) optic nerve, (**c**) frontal cortex, (**d**) visual cortex, and (**e**) cerebellum of NS^−/−^ vs. WT mice. Each dot represents a single protein, identified and quantified, in the experiment. The y-axis represents adjusted −log (*p*-value) while the fold change in abundance (NS^−/−^/WT) is plotted on the x-axis. Vertical blue lines set the nominal cut-off value (1.2 and 0.83) for fold change (±20%) and DEPs outside dashed horizontal lines are considered significant. Down-regulated proteins are represented in blue and up-regulated proteins are shown in red. (**f**) A Venn diagram demonstrating the shared and exclusive DEPs in each region.

As suggested by volcano plots in Figure 3, a total of 39, 46, 285, 697, and 586 proteins were differentially expressed in RE, ON, FC, VC, and CB, respectively, totalizing 1235 unique differentially expressed proteins. Interestingly, the highest number of DEPs was observed in the visual region, followed by CB and FC. Although there were no shared dysregulated proteins among all these five regions, 78 proteins were shared between brain tissues, whereas the RE and ON just presented Haptoglobin (Hp) in common. The visual cortex exhibited the highest number of exclusive DEPs followed by the cerebellum and frontal cortex (Figure 3f). The top 10 differentially dysregulated proteins in all regions of the neuroserpin-deficient mice compared to WT controls are shown in Table 1. Notably, the expression of α-1 Antitrypsin (Serpina1e) from the serpin family shows almost a three-fold increase in the cerebellum, while it shows a decrease in the optic nerve (two-fold) and retina. The complete list of identified proteins for each region as well as their related DEPs are provided in the Supplementary Table S1.

### 3.2. Functional Pathway and Protein–Protein Interaction Analysis Identify Region-Specific Pathways Affected in the Brain and Retina of NS^−/−^ Mice

**Figure 4:**
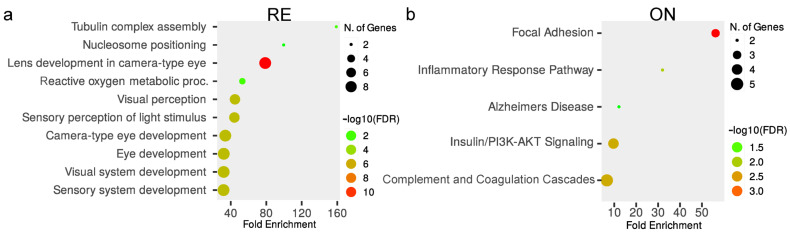

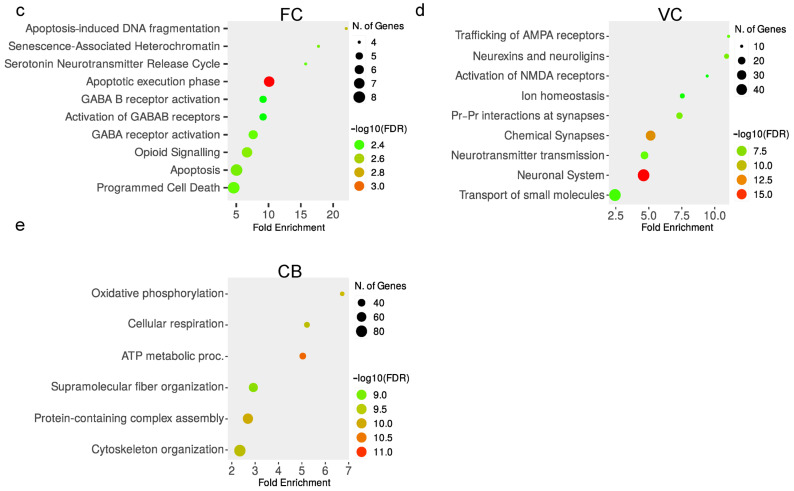
Top 10 significantly (FDR ≤ 0.05) enriched pathways in response to neuroserpin ablation in the (**a**) retina, (**b**) optic nerve, (**c**) frontal cortex, (**d**) visual cortex, and (**e**) cerebellum, sorted by the fold enrichment on the x-axis. Warmer colors show increasing -log10 (FDR) and the size of the circles represent the number of proteins involved in each term.

To better understand the pathways and physiological processes affected after neuroserpin knockout, differentially expressed proteins in each region were subjected to pathway enrichment analysis. The enrichment analysis of DEPs revealed changes in various biological processes in a region-specific pattern. Biochemical pathways associated with nucleosome positioning, visual perception, focal adhesion, complement/coagulation cascade, activation of GABA receptors, apoptosis, glutamate receptors, neurexins/neuroligins, oxidative phosphorylation, and, notably, supramolecular fiber organization showed significant enrichment (FDR < 0.05) in various tissues of NS^−/−^ mice compared to their WT counterparts (Figure 4).

**Figure 5:**
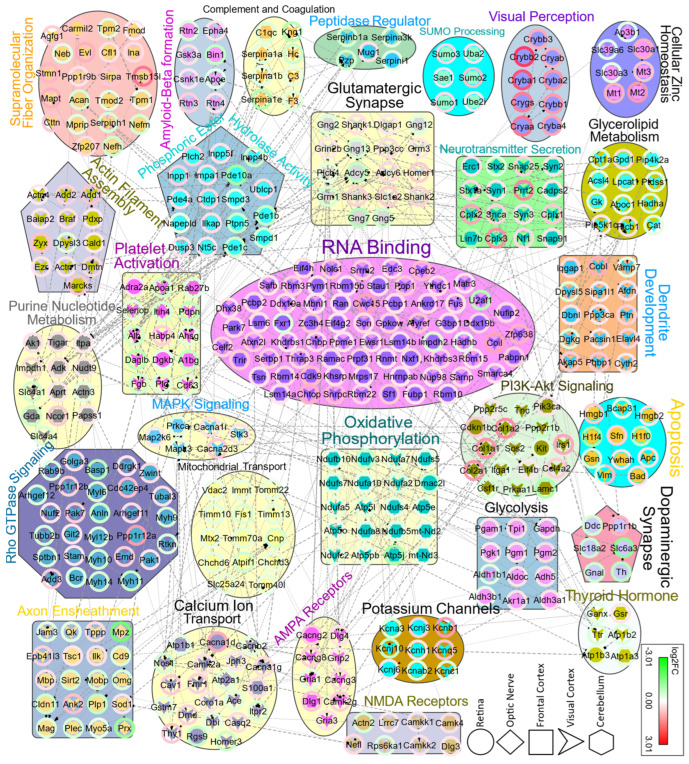
Protein–protein interaction network (PPI) for 1235 pooled DEPs from all regions, analyzed by the Cytoscape stringApp plugin and OmicsVisualizer. Network nodes are labeled using gene symbols. The stringApp plugin was used to assign significantly enriched (FDR < 0.05) BP, MF, and Pathway to clusters, which were then color-coded with pie charts showing the fold changes in DEPs. Only high confidence clusters with a STRING score > 0.7 and nodes ≥ 6 are displayed. Dashed edges represent interactions deriving from experiments; dotted edges indicate interactions based on databases. DEPs originated from RE, ON, FC, VC, and CB are drawn as a circle, rhombus, square, V-shape, and hexagon, respectively.

Generally, GO analysis reveals the classification of differentially expressed proteins and how they might be involved in various processes. To obtain a broader perspective and further explore the interaction between the proteome of each region, we analyzed pooled DEPs for protein–protein interaction (PPI). Differentially expressed proteins across all regions were pooled together into 1235 unique gene symbols and analyzed for network enrichment. We found that most of these DEPs interact with a high confidence (dashed lines indicate interactions derived from experiments, while dotted lines represent interactions based on databases). Interestingly, many pathways and GOs that exhibited dysregulation in each region also appeared in PPI functional enrichment analysis (Figure 5). Moreover, there were novel affected pathways emerging from the pooled DEPs across the tissues. As illustrated in Figure 5, several PPI clusters with assigned enriched terms like RNA binding, axon ensheathment, Rho GTPase signaling, calcium ion transport, PI3K-AKT signaling, amyloid-beta formation and mitochondrial transport emerged. The PPI functional enrichment analysis revealed how dysregulated proteins might interact with other proteins to form complexes, demonstrating how various clusters are interconnected through their constituent proteins across different regions.

### 3.3. Nucleosome Positioning and Visual Perception Are Affected in the Retina of Knockout Mice

Proteomic analysis of the retina revealed 39 proteins to be differently regulated in NS^−/−^ mice compared to controls. (Figure 3a and Supplementary Table S1). Functional enrichment analysis suggests these proteins are mainly related to nucleosome positioning and visual perception (Figure 4a). Out of 10 histone proteins identified in the proteomics experiment, histone H1 (H1f1) and H5 (H1f5) were observed to be down-regulated in KO retina, which highlights the role of Serpini1 in nucleosome positioning (enrichment FDR = 2.1 × 10^−2^). Additionally, nine proteins (Cryaa, Cryab, Cryba1, Cryba2, Cryba4, Crybb1, Crybb2, Crybb3, and Crygs) from alpha, beta, and gamma families of mammalian crystallins were differentially modulated across knockout and controls. All these crystallin members were observed to be up-regulated in the retina and were associated with visual perception (enrichment FDR = 2.7 × 10^−6^).

### 3.4. Optic Nerve Shows Dysregulation of Focal Adhesion and Complement/Coagulation Cascades in the Absence of Serpini1

The optic nerve showed a total of 46 proteins to be differentially regulated in NS^−/−^ mice compared to controls (Figure 3b and Supplementary Table S1). The functional enrichment analysis reveals these proteins are related to the focal adhesion (enrichment FDR = 5.6 × 10^−3^) and complement/coagulation cascade pathways (enrichment FDR = 1.6 × 10^−2^). Focal adhesion-associated proteins including collagen type I alpha 1 chain (Col1a1), collagen type I alpha 2 chain (Col1a2), collagen type II alpha 1 chain (Col2a1), collagen type XII alpha 1 chain (Col12a1), and Bcl2-associated agonist of cell death (Bad) were all observed to be up-regulated in the optic nerves of KO mice at 12 months of age. Complement and coagulation cascade pathways included two members of Serpin family A (Serpina1a and Serpina1e) with a decrease of 0.79 and 0.55-fold, respectively (Figure 4b).

### 3.5. Activation of GABA Receptors and Apoptosis in the Frontal Cortex of NS^−/−^ Mice

The frontal cortex showed a total of 285 proteins to be significantly dysregulated in neuroserpin-deficient mice compared to controls (Figure 3f and Supplementary Table S1). The enrichment analysis revealed two relevant pathways, GABA receptor activation (enrichment FDR = 3.6 × 10^−3^) and apoptosis (enrichment FDR = 3.4 × 10^−3^) are mostly modulated in the FC. Several components of GABA receptor activation were identified (Adcy5, Gnal, Gng7, Gabra4, Kcnj3, and Kcnj6) as differentially expressed in the frontal cortex of KO/WT mice. Proteins like adenylate cyclase 5 (Adcy5), G protein subunit alpha L (Gnal), and G protein subunit gamma 7 (Gng7) showed a significant decrease in abundance, whereas gamma-aminobutyric acid type a receptor subunit alpha 4 (Gabra4) and potassium inwardly rectifying channel subfamily J member 3 (Kcnj3) and 6 (Kcnj6) displayed an up-regulation trend. There were upregulated DEPs assigned to apoptosis pathways (Figure 4c), including microtubule-associated protein tau (Mapt), 14-3-3 eta (Ywhah), adducin 1 (Add1), as well as down-regulated proteins like APC regulator of WNT signaling pathway (Apc) and four members of histone family (H1f0, H1f3, H1f4, and H1f5). Remarkably, our quantitative proteomics dataset revealed a subset of proteins associated with the dopamine neurotransmitter release cycle (Cplx, Slc18a2, Syn1 and Syn3) to be affected in the frontal cortex of KO brains (enrichment FDR = 7.1× 10^−3^).

### 3.6. Enrichment of Glutamate Receptor Activation and Neurexins/Neuroligins in the Visual Cortex

We identified several DEPs associated with glutamate receptor activation and the regulation of neurexins/neuroligins homeostasis in the visual cortex. AMPA (α-amino-3-hydroxy-5-methyl-4-isoxazolepropionic acid) and NMDA (N-methyl-D-aspartate) receptors are two types of glutamate receptors that play a crucial role in neurotransmission within the CNS. Neurexins and neuroligins play crucial roles in synapse communication and are presynaptic and postsynaptic cell adhesion molecules, respectively. In our dataset, discs large MAGUK scaffold protein 4 (Dlg4) was upregulated and shared between all these three enriched pathways. Additionally, alpha and gamma chains of calcium/calmodulin-dependent protein kinase II (Camk2a and Camk2g) as well as discs large MAGUK scaffold protein 1 (Dlg1) were upregulated and shared between both AMPA and NMDA receptors. The rest of the proteins associated with AMPA receptor activation were Akap5, Cacng3, Cacng8, Gria1, Gria3, and Grip2. Activation of NMDA receptors included other DEPs like Camkk1, Camkk2, Lrrc7, Nefl, Nrgn, and Rps6ka1. Lastly, proteins like Dlgap1, Homer3, Homer1, Grm1, Shank3, Epb41l3, Shank2, Shank1, Dlgap3, Dlgap2, and Dlgap4, involved in the homeostasis of neurexins/neuroligins, were observed to be dysregulated in the NS^−/−^ visual cortex.

### 3.7. Oxidative Phosphorylation and Supramolecular Fiber Organization Are Affected in the Cerebellum of NS^−/−^ Mice

We quantified 22 DEPs associated with oxidative phosphorylation (enrichment FDR = 2.5 × 10^−10^) in the NS^−/−^ cerebellum, including members of the ATP Synthase family (Atp5e, Atp5j, Atp5k, and Atp5o), subunits of cytochrome c oxidase (Cox4i2, Cox5a, Cox6c, Cox7a1, Cox7a2, Cox7b, and Cox7c), several subunits of ubiquinone oxidoreductase (Ndufa10, Ndufa7, Ndufa8, Ndufs7, and Ndufv3), and two components of ubiquinol-cytochrome c reductase (Uqcrb and Uqcrh). Another prominent pathway that was significantly affected in the cerebellum was supramolecular fiber organization (enrichment FDR = 7.3 × 10^−10^) with 55 DEPs. Several proteins from this GO identified to be up-regulated, including Adducin 1 and 3 (Add1 and Add3) and microtubule-associated protein 1s and tau (Map2 and Mapt), while there was a down-regulation in other interesting proteins like heavy, medium, and light chains of neurofilaments (Nefh, Nefm, and Nefl), myosins (Myh10, Myh11, Myo1d, and Myo5a), and tropomyosin family (Tpm1 and Tpm2).

### 3.8. Validation of Quantitative MS Data Using Western Blotting

**Figure 6:**
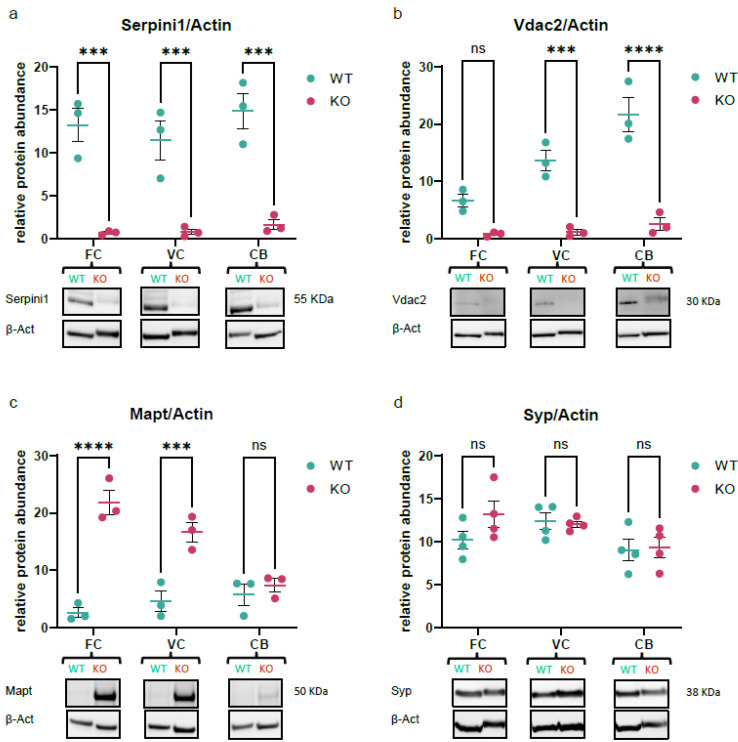
Validation of key proteins quantified in TMT proteomics experiment. Western blot quantifications for (**a**) Serpini1, (**b**) Vdac2, (**c**) Mapt, and (**d**) synaptophysin, normalized to expression levels of β-Actin in the frontal cortex, visual cortex, and cerebellum regions from NS^−/−^ versus WT mice. Data were plotted as mean ± SEM, N ≥ 3 (ns = *p* > 0.05, *** *p* < 0.001, **** *p* < 0.0001, ANOVA).

We performed Western blotting on brain tissues to orthogonally verify observed alterations in protein abundance suggested by quantitative mass spectrometric analysis. This provides additional evidence for the effects of neuroserpin ablation and validates the methodological repertoire of the study. To validate the network of dysregulated proteins, we nominated three proteins, from various enriched Pr–Pr clusters, each with a distinct dysregulation trend (down-regulated, up-regulated, and unchanged), to serve as Western blot candidates. We included the Serpini1 protein itself to characterize the animal model as well. As shown in Figure 6a, all three brain regions showed ablation of neuroserpin which appeared within the expected molecular weight, confirming the absence of a functional Serpini1 in brain regions. Western blotting data suggested the down-regulation of Vdac2 in the VC and CB, consistent with the quantitative proteomic experiments (Figure 6b). Intriguingly, tau protein (Mapt) was up-regulated in all three brain regions according to TMT-proteomics data, but Western blotting only confirmed an increase in FC and VC, not detecting significant changes in CB (Figure 6c). Finally, the lack of changes in synaptophysin (Syp) protein levels across all brain regions in knockout mice compared to controls adds further weight to the consistency of the findings identified in the proteomics analysis (Figure 6d). Together, orthogonal antibody-based validation confirmed three key proteins having distinct trends to be similarly affected in knockout mouse brains with a negligible exception.

## 4. Discussion

In the current study, we used quantitative mass spectrometry-based proteomics with TMT labeling to investigate the region-based proteome changes in the retina and brain tissues in Serpini1 knockout mice. We found different regional dysregulation of biological pathways including visual perception, apoptosis, glutamate receptor activation, and supramolecular fiber organization. While isobaric labeling using tandem mass tags is considered a valuable and precise technique in quantitative proteomics, there are limitations in analyzing the data from multiple batches of TMT experiments [46]. As many technical challenges arise when data from multiple separate multiplex TMT batches are integrated, we decided to use a MS2-based TMT quantitation approach [47,48]. Furthermore, although each region requires customized chromatographic and MS acquisition methods to increase the coverage and quantification, we used the same running conditions to minimize the batch-effect and maximize protein comparison between the regions. There are other inherent limitations in our work, primarily due to employing a bottom–up proteomics approach, which involves proteolytic cleavage of proteins into peptides. While peptides result in better separation, ionization, and fragmentation in a predictable manner, the link between identified peptides and their parent proteins is lost during the enzymatic digestion step [49,50]. Thus, data derived from short peptides are not sufficient for the detection and quantification of proteoforms which contribute to the complexity of biological systems and allow proteins to perform specific functions in different cellular contexts [51,52]. As an alternative, top–down proteomics explore proteins in their intact forms and is better suited for the characterization of proteoforms [53].

When exploring the phenotype associated with a knockout gene, a key consideration is that the specific protein has been absent since conception, which may make it complicated to distinguish perturbations due to the gene knockout itself from changes caused by biological compensation for that missing protein [54]. We hypothesized that some members of the Serpin super-family can compensate for the Serpini1 depletion, and their expression would increase in the analyzed regions. We observed the up-regulation of three members of the serpin family (Serpina1a, Serpina1e, and Serpina3k) in the cerebellum, and, to our surprise, the down-regulation of different serpins in the rest of the analyzed regions (Supplementary Table S1). These observations indicate that the loss of Serpini1 expression does not necessarily lead to a compensatory rise in other serpins at a global level in the nervous system. Counterintuitively, while neuroserpin inhibits tPA, it has been previously described that our knockout model does not exhibit increased proteolytic activity of tPA [32,55]; therefore, the observed proteome changes are in part tPA-independent effects of neuroserpin [56].

Further, in our proteome analysis of NS^−/−^/WT mice, we found that the retina and optic nerve did not yield an equal number of DEPs similar to brain regions; only 39 and 46 dysregulated proteins were quantified in RE and ON, respectively. This might be due to the fact that this serpin is mainly thought to mediate synaptic plasticity in the brain [12,57], while the retina and optic nerve are primarily responsible for processing and sending light sensory stimuli to higher visual centers. Moreover, Serpini1 is abundantly expressed in the brain as secreted vesicles [12,58,59], which makes the brain the major niche for fulfilling its native functions in terms of various biological and molecular processes. Such spatial distribution potentially extends to the interacting partners/inhibitors, post-translational modifications (PTMs), transcriptomics, and epigenetic landscapes which ultimately result in a region-specific loss of function.

This is the first high-resolution study that explores the proteome changes in response to neuroserpin ablation in a region-wise manner; regions which are considered to be associated with diverse functions, such as light detection [60], transmission of visual signals [61], behavior [62], visual Information processing [63] and motor coordination [64]. The diverse functions of these areas were in part reflected in the proteomic results, where each region revealed specific enriched pathways, such as visual perception in the RE, focal adhesion in the ON, activation of GABA receptors in the FC, and NMDA receptors in the VC. There has only been one label-free quantification (LFQ) proteomics study previously conducted on the isolated synaptosomes from the neocortex of adult neuroserpin-deficient mice, which successfully quantified 1268 proteins in at least three out of four biological replicates. However, no significant differentially expressed proteins in the synaptic proteome of neuroserpin-deficient mice were identified, probably due to a strict fold change cutoff threshold [31].

The mouse brain is an intricately interconnected network of thousands of neural cell types [65,66], with spatially distinct and yet functionally linked regions that exhibit precise regulation at the protein expression level [67]. Proteomic analyses of pooled DEPs extracted from all areas suggested novel enriched biochemical networks that have not been previously described for neuroserpin, including functions like RNA binding and axon ensheathment. Nevertheless, some of these Pr–Pr enriched pathways have been reported earlier. For PI3K-AKT signaling, a previously published report has highlighted the protective role of Serpini1 against exogenous hydrogen peroxide, via AKT pathway activation [68]. Similarly, thyroid hormone is thought to bind to its Thr1β receptor and increase neuroserpin expression levels in AD [69]. Here, we detected the dysregulation of thyroid hormone synthesis, mostly in the visual cortex of KO animals, though mediated through other proteins (Figure 5). Interestingly, our interactome analysis suggests the perturbation of proteins involved in amyloid-beta and tau protein regulation which play key roles in neurodegenerative disorders such as AD [70].

In order to validate our findings, we confirmed three DEPs displaying distinct regulation trends using an antibody-based approach. Western blot analysis for voltage-dependent anion-selective channel protein 2 (Vdac2) confirmed the down-regulation trend suggested by TMT proteomics in the VC and CB of knockout mice (Figure 6b). Mitochondrial homeostasis is critical to the function of neurons [71,72], where Vdac2 is a key regulator of metabolite flux across the outer membrane [73]. This porin promotes mitophagy in early brain injury *in vivo* [74], protects neurons against BAK-dependent mitochondrial apoptosis [75], and has been reported to be protective during aging [76,77]. Given the downregulation of Vdac2 in KO regions, it is likely that neuroserpin exerts a neuroprotective effect through controlling Vdac2 expression levels.

Another candidate that was validated using Western blotting was tau protein (Mapt), which showed upregulation in all brain regions and Western blotting confirmed the trend in the FC and VC (Figure 6c). Hyperphosphorylated tau protein in the form of neurofibrillary tangles (NFTs) is a hallmark of AD pathology [78,79], in which the neuroserpin level was reported to be associated with tau protein phosphorylation in the cerebrospinal fluid (CSF) [80]. The increase in tau expression in KO mice suggests that neuroserpin is potentially a regulator of tau expression in the FC and VC, but its precise mechanism still needs further study. Since the monoclonal anti-tau antibody (tau46) we used was predicted to detect all six proteoforms of this protein, identifying the exact isoform that is modulated by neuroserpin needs further investigation. Lastly, we checked the expression levels of synaptophysin (Syp) which is a well-known marker of presynaptic plasticity and synaptogenesis [81]. Previous works have reported that synaptophysin expression is unaffected in the NS^−/−^ retina [20] or hippocampus [14], a similar pattern we observed in our Western blot quantifications (Figure 6d) and MS data. To address the discrepancies between MS data and Western blotting for tau protein in the cerebellum, it is helpful to acknowledge that antibody-based techniques, in general, might not exactly recapitulate the same observations suggested by MS methods [82]. It is known that differences in protein expression levels observed using different methods may arise from various technical factors, and it is crucial to carefully consider the limitations and specific conditions of each analytical method when interpreting results [83].

## 5. Conclusions

In summary, our study delineated region- and tissue-specific changes in protein expression in the CNS in the absence of Serpini1. This work improves our current understanding of neuroserpin-regulated interactions or the biological processes in the brain and parts of the visual pathway. In order to gain further insights, it will be helpful to integrate data derived from individual omics technologies (genomics, transcriptomics, proteomics, and metabolomics) [84]. The integration of the proteomics data with other high-throughput data can provide complementary insights into the biological functions of Serpini1, both in normal physiology and pathological conditions. Consequently, we expect that our findings will improve our understanding of neuroserpin functions in the nervous system and better harness its potential neuroprotective mechanisms in the future.

## Figures and Tables

**Table 1:** Top 10 dysregulated proteins in five regions with *p*-value lower than 0.05. The red and blue colors represent up- and down-regulated proteins, respectively.

	Retina		Optic Nerve		Frontal Cortex		Visual Cortex		Cerebellum	
	Gene ID	FC	Gene ID	FC	Gene ID	FC	Gene ID	FC	Gene ID	FC
**Up-Reg** **(NS^−/−^ vs. WT)**	Crybb2	5.01	Col1a2	2.58	Sp8	2.47	Nrgn	4.42	Serpina1e	2.94
	Esp6	1.82	Tinagl1	1.82	Ppp1r12a	2.22	Psd3	3.46	Zdhhc5	2.47
	Nt5dc3	1.76	Pdzd8	1.33	Crtc1	2.04	Tmsb15l	3.06	Tnrc6b	2.34
	Zfyve19	1.65	Psd2	1.32	Slc30a1	1.90	Rnf25	2.73	Mt2	2.28
	Sf1	1.53	Cep170b	1.29	Psd3	1.87	Prrt2	2.62	Exosc9	2.22
**Down-Reg** **(NS^−/−^ vs. WT)**	Hp	0.70	Mpz	0.33	Pde10a	0.47	Plscr4	0.28	Cox4i2	0.12
	Serpina1e	0.74	Prx	0.44	HBB1	0.53	Calb2	0.41	Fxyd7	0.39
	H1-5	0.76	Serpina1e	0.55	Ankrd63	0.55	Pde10a	0.50	Atp5pf	0.50
	Ca1	0.81	Hp	0.59	Gng7	0.56	Igh	0.52	Igh	0.50
	Tagln	0.81	Arhgef10	0.72	Penk	0.57	Ca1	0.52	H1f4	0.50

## Data Availability

Proteomics data are available via ProteomeXchange with the identifier PXD046873.

## Supplementary Materials

The following supporting information can be downloaded at https://www.mdpi.com/article/10.3390/proteomes12010007/s1, Figure S1: Principal component analysis (PCA) analysis of KO/WT samples for each region; Table S1: Complete list of proteins, with relevant statistics as well as differentially expressed proteins in each region.
