# Supplementary figures and images for: Quantitative Proteomics Reveal Region-Specific Alterations in Neuroserpin-Deficient Mouse Brain and Retina: Insights into Serpini1 Function
Source: Proteomes. 2024 Mar 14;12(1):7. doi: 10.3390/proteomes12010007 (PMC10975625; doi:10.3390/proteomes12010007)

## Slide 1
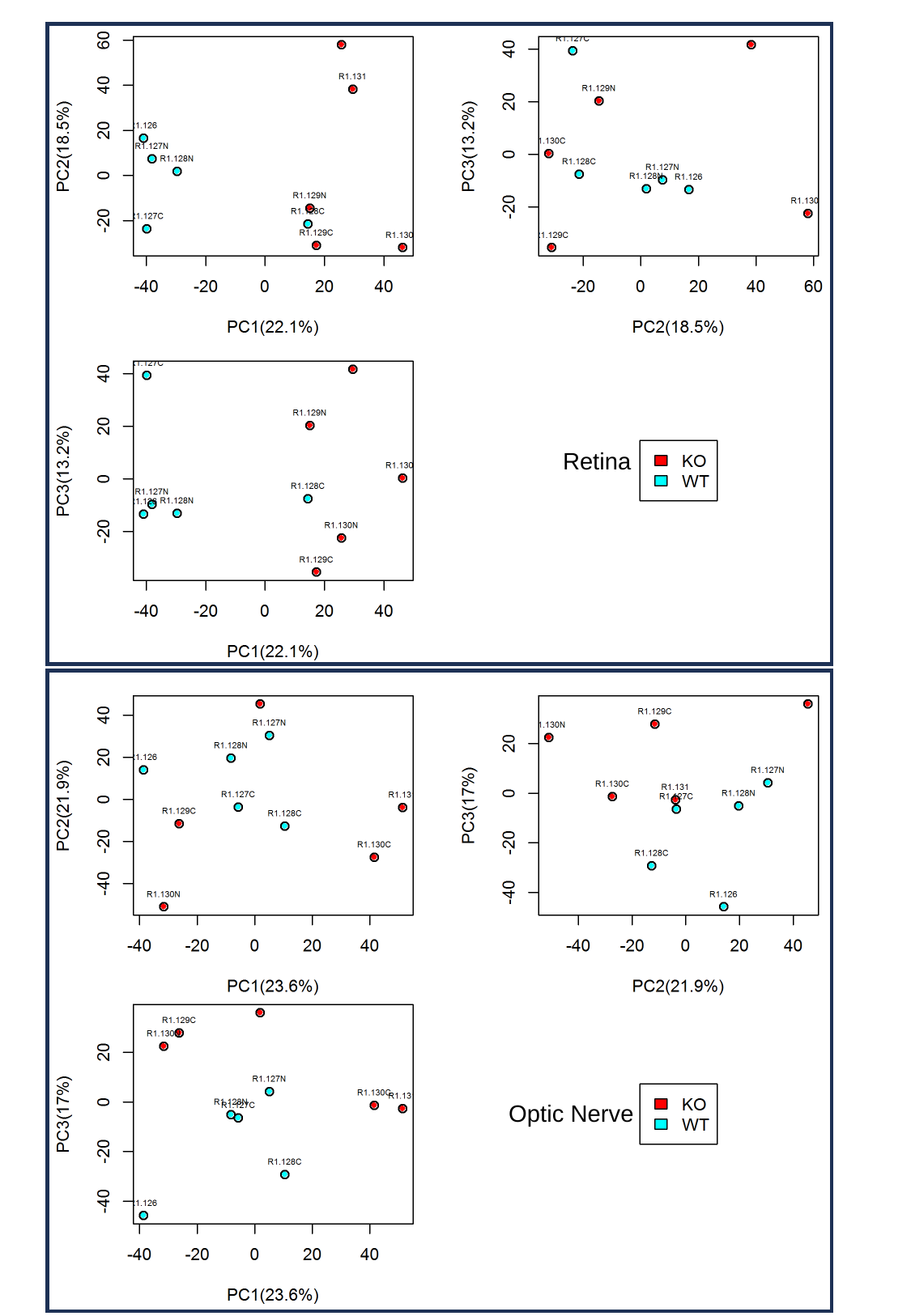

Retina
Optic Nerve

## Slide 2
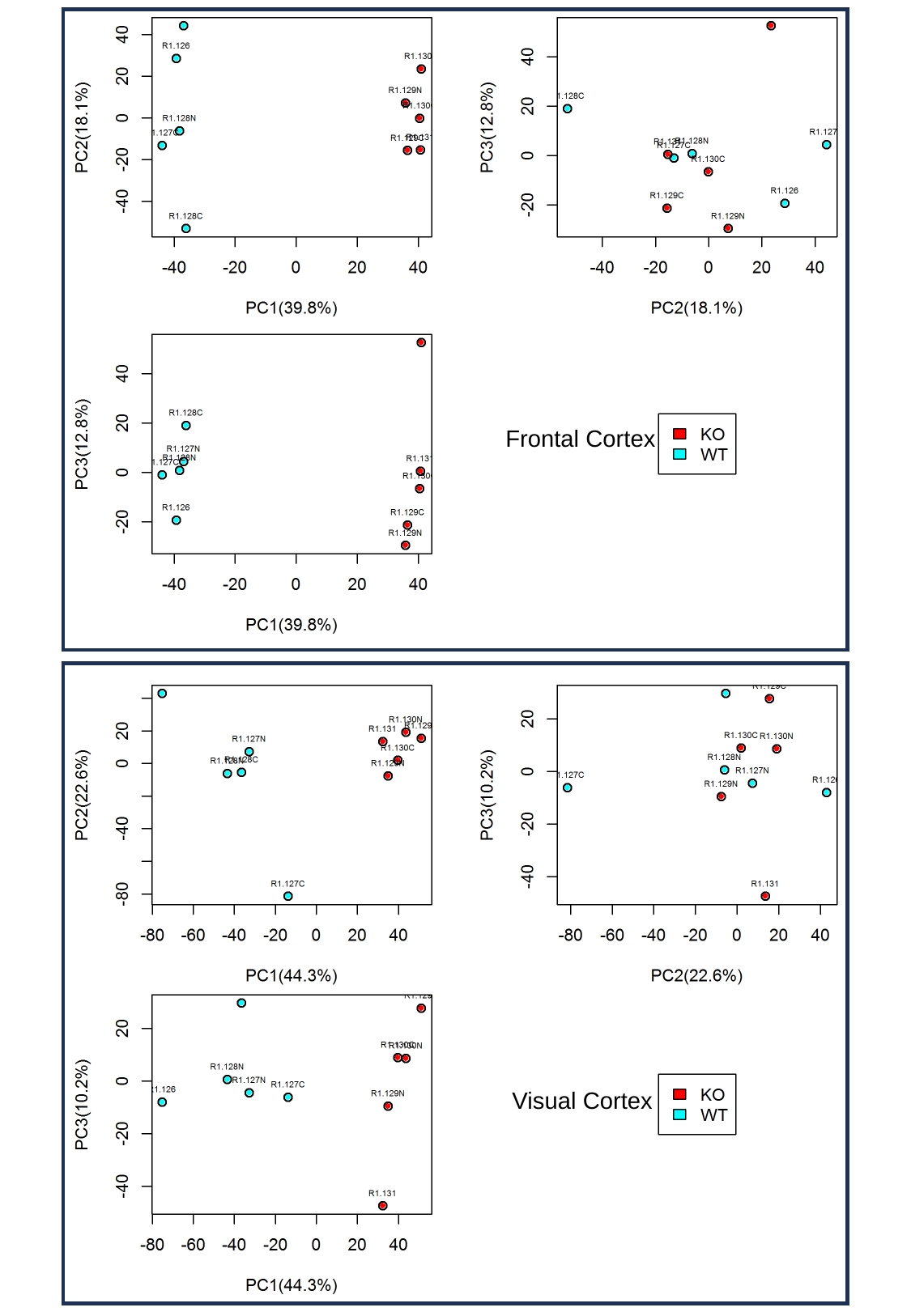

Frontal Cortex
Visual Cortex

## Slide 3
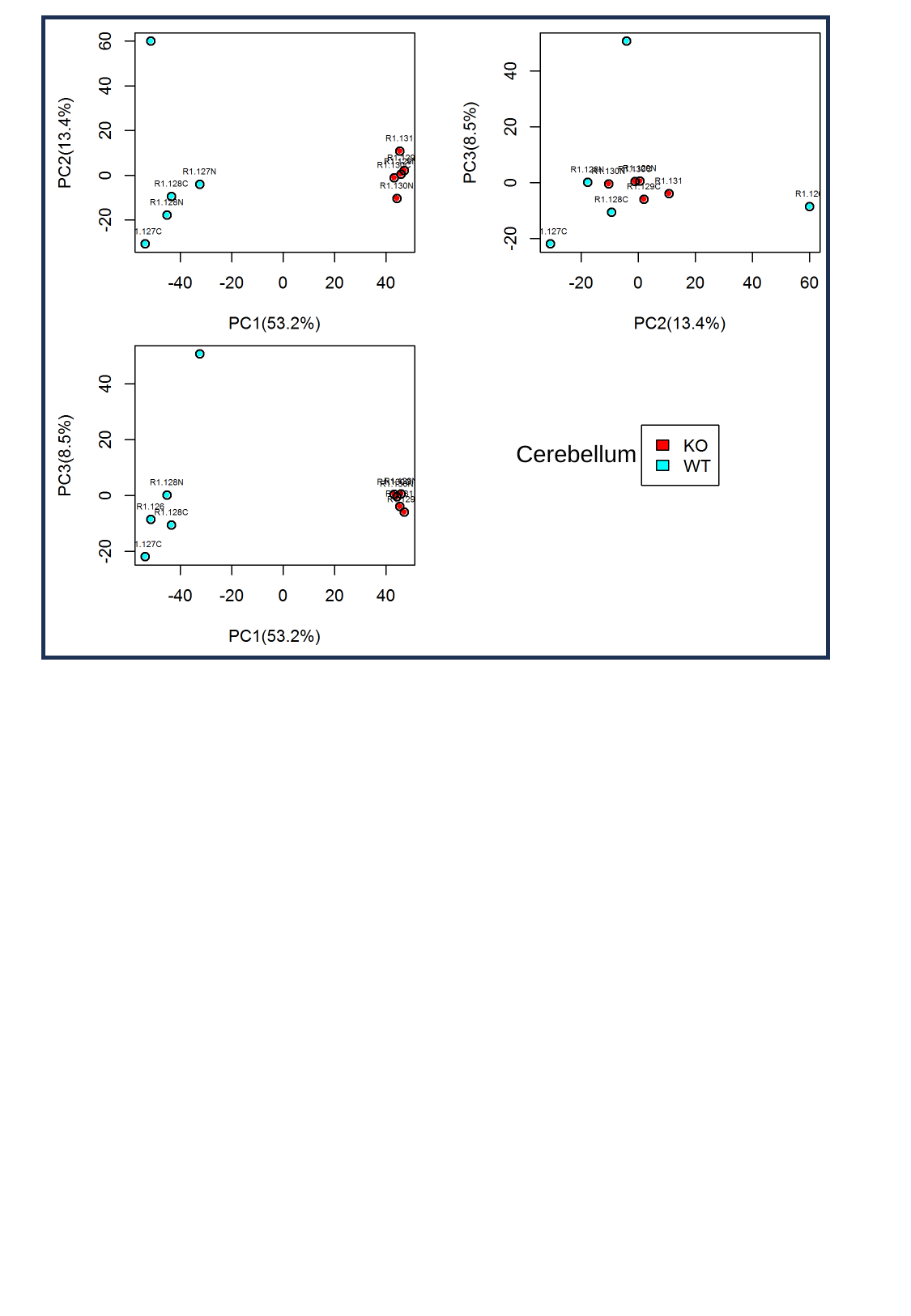

Cerebellum

Supplement: Supplementary file 1 [file proteomes-12-00007-s001.zip › Supp Figure S1.pptx]
